# Obituary

**Published:** 1895-01

**Authors:** 


OBITUARY.—FRANK ARDARY, Jr., V. S., died June 2, 1894, after a brief illness of typhoid fever complicated with pneumonia. He graduated at the Christmas examination of the Ontario Veterinary College in 1883; then returned to his native city, Pittsburg, Pa., and soon established a lucrative practice; then with his brother, R. W., equipped and managed the largest veterinary hospital in the country. His genial disposition and kindly nature endeared him to a large number of friends. He was the leading veterinarian in the city, and very popular with all classes, and ranked high in Masonic circles. Unfortunately, he took no interest in veterinary circles outside of his own business, and never joined our societies.

